# Supplementary material for: The INO80 Chromatin Remodeling Complex Regulates Histone H2A.Z Mobility and the G1‐S Transition in Oligodendrocyte Precursors
Source: Glia. 2025 Feb 28;73(6):1307–23. doi: 10.1002/glia.70006 (PMC12012327; doi:10.1002/glia.70006)
Supplement: Supplementary file 1 — Data S1. Supporting Information. [file GLIA-73-1307-s001.docx]

**SUPPLEMENTAL DATA**

Supplementary Figures S1-S3 and Supplementary Table 1 for:

_______________________________________

**The INO80 chromatin remodeling complex regulates histone H2A.Z mobility and the G1-S transition in oligodendrocyte precursors**

# Jordan L Wright ^#^, Yi Jiang, Stuart G Nayar, Huiliang Li * and William D Richardson*^, §^

Wolfson Institute for Biomedical Research, University College London, Gower Street, London WC1E 6BT, United Kingdom

^#^ current address: Murdoch Children’s Research Institute, Royal Children’s Hospital, Flemington Road, Parkville, Victoria 3052, Australia

* corresponding authors ^§^ lead author

__________________________________________________


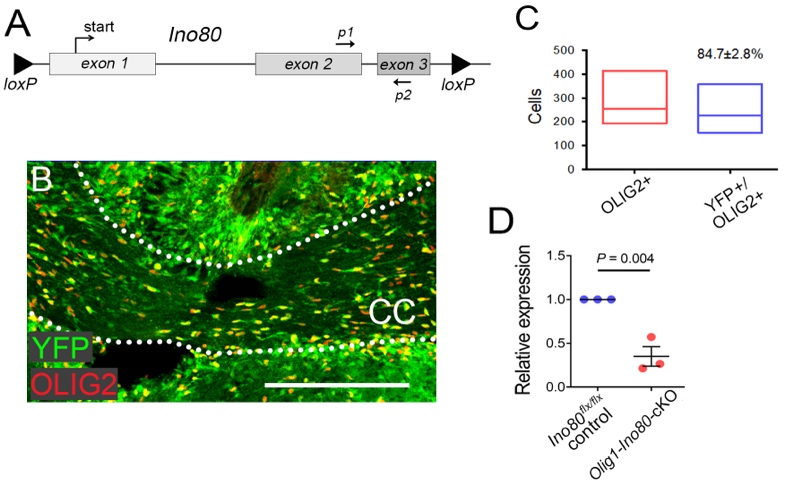


**Supplementary Figure S1.**

**Recombination efficiency in *Ino80-*cKO mice.**

(**A**) Positions of *loxP* sites at the modified *Ino80^flx^* loci in transgenic mice. Also shown are the relative positions of PCR primers (P1, P2) used to assess Cre-mediated recombination in reverse transcription PCR (rtPCR) assays of purified mRNA. (**B**) Fluorescence image of the corpus callosum (CC, dotted lines) from a P7 *Olig1-Cre: Rosa26-YFP* mouse immunolabelled for GFP and OLIG2; double-labelling for OLIG2 and YFP reflects Cre-mediated recombination of the *Rosa26-YFP* reporter transgene in OL lineage cells. (**C**) Quantification of OLIG2^+^ and YFP^+^/OLIG2^+^ cells in P7 *Olig1-Cre: Rosa26-YFP* mice; recombination efficiency is ~85% based on YFP expression. (**D**) Quantitative RT-PCR of RNA from OPs immuno-purified from P7 *Olig1-Ino80-*cKO and *Ino80^flx/flx^* control brains, using primers (*p1, p2*) positioned as in (**A**) suggests that CRE recombination efficiency is ~75%. Error bars represent mean ± s.e.m. Statistical significance based on Student’s t-test. Scale bar: 100 µm (**B**).


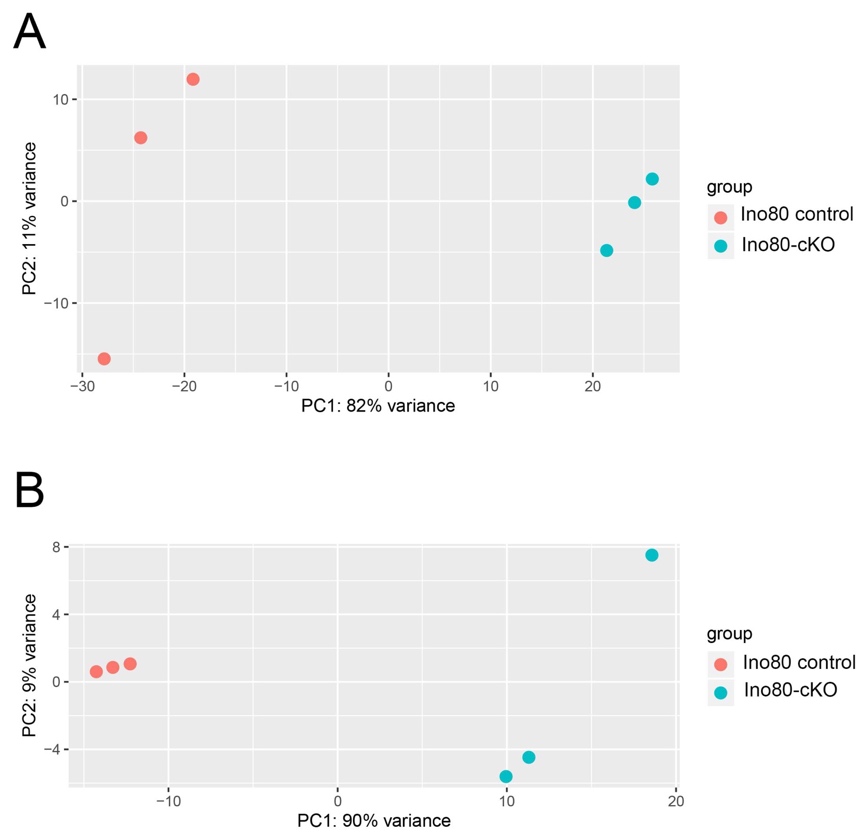


**Supplementary Figure S2.**

**PCA plots of RNAseq and ATACseq datasets.**

PCA analysis of (**A**) RNAseq datasets and (**B**) ATACseq datasets from *Ino80*-cKO (green) and *Ino80* control (red) OP replicates.


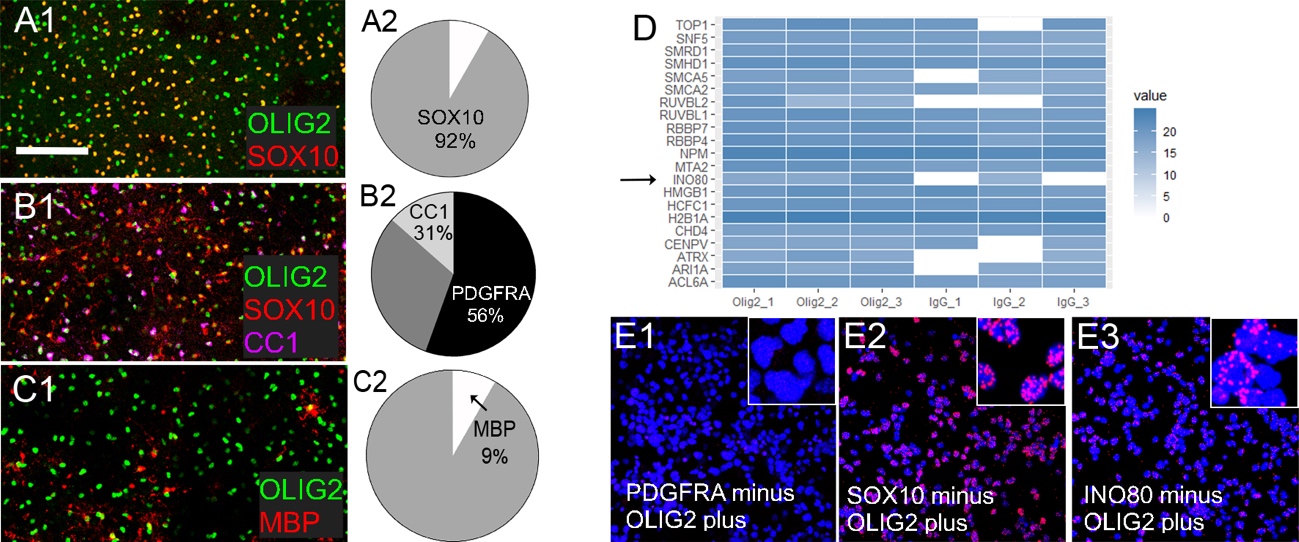


**Supplementary Figure S3. Protein-protein interactions between OLIG2 and components of chromatin remodeling complexes.**

Monolayer cultures were established from dissociated mouse E13.5 cortical hemispheres (neural stem cells, NSCs) and cultured in medium containing thyroid hormone (TH) to induce OL differentiation for 5 days before immuno-labelling for (**A1**) OLIG2 and Sox10, (**B1**) OLIG2, PDGFRA and CC1 antigen and (**C1**) OLIG2 and MBP. Pie charts illustrate the proportions of OLIG2^+^ cells that co-labelled with Sox10 (**A2**), Pdgfra and CC1 (**B2**) and MBP (**C2**). (**D**) Mass spectrometry was carried out on immuno-precipitates (IPs) from 5-day differentiated NSC-derived cultures. Around 175 proteins were identified that were >2-fold more abundant in all three replicate anti-OLIG2 IPs compared to negative control IgG Ips performed under identical conditions. Of these, 22 (~12%) were known components of chromatin remodelling complexes (CRCs), including INO80 (arrow). (**E**) Proximity ligation assays provide further evidence of a close physical interaction between INO80 and OLIG2 in cultured OPs. Pdgfra-minus + OLIG2-plus probes provided a negative control (**E1**) and SOX10-minus + OLIG2-plus a positive control (**E2**). Red puncta indicate sites of close proximity indicating physical interactions between OLIG2 and Sox10 (**E2**), and between OLIG2 and INO80 (**E3**). Magnified images are inset in **E1**-**E3.** Scale bar (**A1-C1**, **E1-E3**): 100 µm.

**
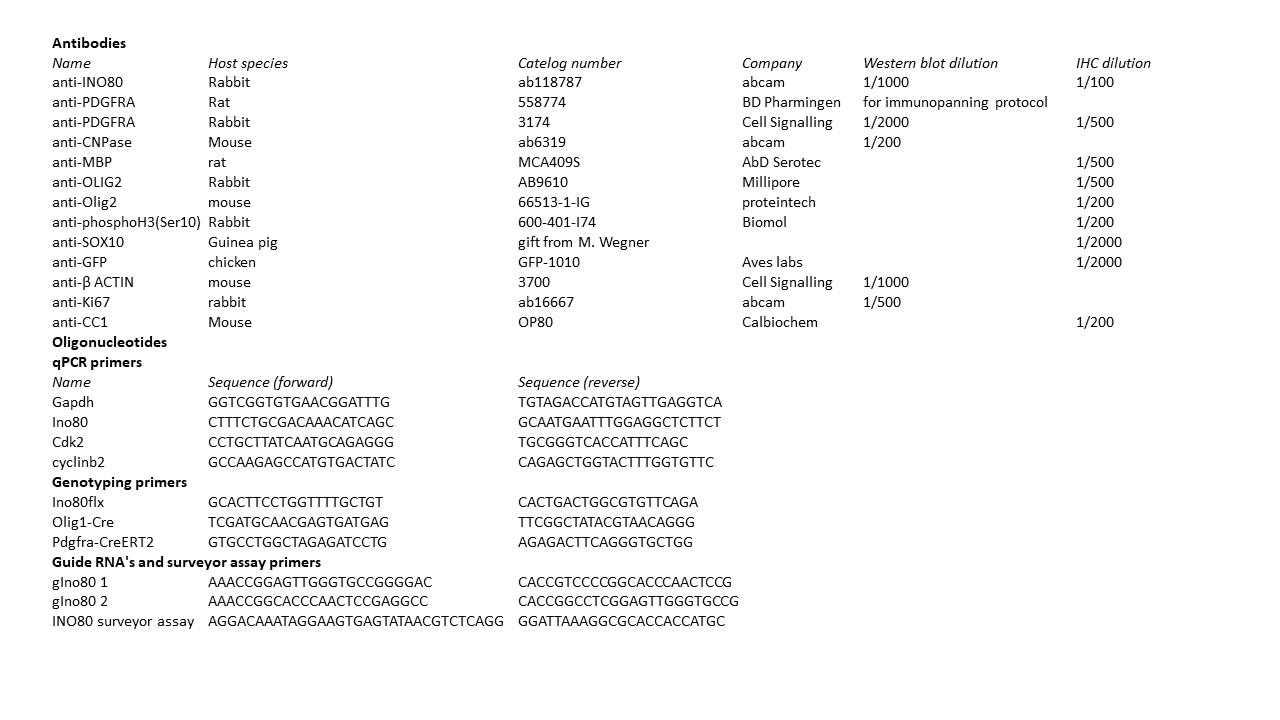
**

**Supplementary Table 1.** List of primary antibodies and DNA oligonucleotides

end
